# Personalized Music Listening and Autobiographical Narration in Nursing Home Residents: Linguistic and Qualitative Findings from a Pilot Study

**DOI:** 10.3390/bs16050810

**Published:** 2026-05-19

**Authors:** Chiara Rossi, Fabio Frisone, Francesca De Salve, Sophia Zanoletti, Paolo Caneva, Matteo Brazzelli, Lorenzo Antichi, Chiara Pupillo, Giuseppe Riva, Osmano Oasi, Barbara Colombo

**Affiliations:** 1 Department of Human Sciences, Guglielmo Marconi University, 00193 Rome, Italy; c.rossi@unimarconi.it; 2 Humane Technology Lab (HTLab), Università Cattolica del Sacro Cuore, Largo Gemelli, 1, 20123 Milan, Italy; fabio.frisone@unicatt.it (F.F.); lorenzo.antichi@unicatt.it (L.A.); chiara.pupillo@phd.unipi.it (C.P.); giuseppe.riva@unicatt.it (G.R.); 3 Department of Theology, Philosophy and Human Sciences, Sophia University Institute, Via di S. Vito, 28, Figline e Incisa Valdarno, 50064 Florence, Italy; 4 Department of Psychology, Università Cattolica del Sacro Cuore, Largo Gemelli, 1, 20123 Milan, Italy; francesca.desalve@unicatt.it (F.D.S.); sophiazanoletti@alice.it (S.Z.); osmano.oasi@unicatt.it (O.O.); 5 Piccolo Cottolengo Don Orione, 20831 Seregno, Italy; paolo.caneva@conservatorioverona.it (P.C.); matteo.brazzelli@orioneseregno.it (M.B.); 6 Department of Computer Science, University of Pisa, Largo B. Pontecorvo, 3, 56127 Pisa, Italy; 7 Applied Technology for Neuro-Psychology Laboratory, IRCCS Istituto Auxologico Italiano, Via Magnasco 2, 20149 Milan, Italy; 8 School of Psychology, Fielding Graduate University, 2020 De La Vina Street, Santa Barbara, CA 93105, USA

**Keywords:** autobiographical memory, personalized music intervention, narrative process, linguistic analysis, nursing home residents

## Abstract

Autobiographical memory plays a central role in identity continuity, narrative functioning, and psychological well-being in later life. In nursing home residents, however, reduced environmental stimulation, cognitive vulnerability, and limited opportunities for self-expression may compromise autobiographical engagement. Music, as an emotionally salient and personally meaningful cue, may facilitate memory retrieval through affective and self-referential mechanisms. This exploratory pilot study examined whether personalized music listening influences the productivity and linguistic features of autobiographical narration in nursing home residents. Eleven older adults completed one baseline autobiographical recall session without music and three weekly music-assisted sessions focused on different life periods. Narratives were transcribed and analyzed using LIWC-22. Within-subject differences were tested with Wilcoxon signed-rank analyses. In addition, an exploratory qualitative paper-and-pencil analysis was conducted to identify recurrent narrative and experiential patterns in the music-assisted accounts. Music-assisted recall was associated with higher total word count compared with baseline, although this difference should be interpreted cautiously given the asymmetry between the single-session baseline and the three-session post-intervention format. No significant changes emerged in positive or negative emotion words. Qualitative observations of the music-assisted narratives highlighted recurrent features including vivid autobiographical scenes, references to meaningful social identities and former life roles, and emotionally salient communication. These preliminary findings suggest that personalized music may support autobiographical recall by increasing verbal output during narration and by facilitating meaningful self-expression and relational communication in later life. Larger controlled studies are needed to clarify its role in supporting autobiographical narrative processes in nursing home older adults.

## 1. Introduction

Maintaining a coherent sense of self across the lifespan is a central psychological challenge of aging, particularly when cognitive resources decline and environmental stimulation is reduced. In these contexts, interventions that can support identity continuity and emotional regulation by promoting autobiographical remembering and narrative engagement may be especially relevant.

Autobiographical memory (AM) has been described as an “archive of the self”, integrating personal events, autobiographical knowledge, and self-relevant meanings that sustain perceived self-continuity over time (Brewer, 1988). AM contributes to psychological functioning by linking experience to present goals and self-understanding, thereby supporting identity coherence and well-being in later life (Addis & Tippett, 2004; Simpson et al., 2023). Emotional salience is a key feature of autobiographical memory, and reminiscence processes have been associated with greater well-being and social connectedness in older age (Talarico et al., 2004; Wildschut et al., 2006; O’Rourke et al., 2011).

According to the Self-Memory System, AM is hierarchically organized, with specific episodic events embedded within broader life-period representations. (Conway & Pleydell-Pearce, 2000; Conway, 2005). Complementing this view, narrative identity frameworks emphasize that individuals construct an evolving life story through which memories are organized into a coherent sense of self (McAdams & Olson, 2010; Vanderveren et al., 2017). Thus, in later life, well-being may depend not only on retrieving memories, but also on narrating them in meaningful ways. Beyond memory retrieval itself, the phenomenological qualities of autobiographical narration (such as vividness, specificity, emotional depth, and the capacity to communicate lived experience to others) may be especially relevant in institutional contexts, where opportunities for identity expression and relational exchange can become restricted (Gauer et al., 2010).

Research on aging has traditionally focused on autobiographical memory specificity (Williams, 1996; Watkins & Teasdale, 2001; Wilson & Gregory, 2018). However, growing evidence suggests that aging also affects the linguistic expression of autobiographical narratives. Compared with younger adults, older adults may produce fewer episodic details and rely more on semantic or generalized information (Levine et al., 2002). Aging has also been associated with changes in lexical diversity, grammatical complexity, and discourse organization (Kemper et al., 2001; Burke & Shafto, 2004). These linguistic dimensions can be examined using computational methods. Linguistic Inquiry and Word Count (LIWC) is a validated dictionary-based text analysis tool that quantifies psychologically meaningful word categories (Tausczik & Pennebaker, 2010; Pennebaker et al., 2015; Boyd et al., 2022). LIWC categories have been widely used as indirect indicators of emotional, cognitive, and interpersonal processes expressed through language. For example, the cognitive processes category includes terms related to causation, insight, discrepancy, and certainty, whereas exclusion words may reflect contrastive differentiation or the organization of alternative perspectives within narratives. Importantly, these categories should be interpreted as probabilistic linguistic markers rather than direct measures of internal psychological states. More recent natural language processing models (e.g., BERT) may further capture contextual meaning and discourse-level patterns, offering complementary approaches for future research (Devlin et al., 2019; Fraser et al., 2016). Nevertheless, computational indices should ideally be complemented by qualitative examination of how autobiographical experiences are narrated, interpreted, and shared.

These issues may be especially relevant in nursing home settings. Although residential care can provide safety and support, it may also reduce opportunities for autonomy, agency, and narrative self-expression (de Medeiros et al., 2020; El Haj et al., 2024). Cognitive decline and depressive symptoms are common in these populations and may further compromise autobiographical specificity and narrative engagement (Fivush et al., 2017; Gonzalez-Colaço Harmand et al., 2014; Kim et al., 2025). This makes non-pharmacological interventions aimed at supporting memory, identity, and well-being particularly relevant.

In this context, music is a particularly promising autobiographical cue. Music is emotionally salient, personally meaningful, and strongly linked to memory networks (Blood & Zatorre, 2001; Koelsch, 2014). Music-evoked autobiographical memories are typically vivid and self-relevant and may reduce the executive demands of retrieval by providing a structured cue that efficiently activates associative networks linking emotion and memory (El Haj et al., 2012). They may also promote scene reconstruction, emotional communication, and re-engagement with personally meaningful roles and relationships (Rossi et al., 2024a; Jakubowski & Francini, 2023).

Personalized music listening has shown beneficial effects on mood and behavioral symptoms in older adults, including in dementia care and residential settings (Wall & Duffy, 2010; Bellelli et al., 2012; Gerdner, 2012). Its effectiveness likely depends on selecting familiar and autobiographically meaningful songs linked to identity-forming life periods (Rubin et al., 1986; Janata et al., 2007; Koppel & Rubin, 2016; Jakubowski & Eerola, 2022).

Building on this background, the present exploratory pilot study examined whether personalized music listening influences narrative productivity and linguistic features of autobiographical recall in nursing home residents. We investigated whether music-assisted retrieval would enhance narrative productivity and promote linguistic markers potentially associated with cognitive elaboration and integrative processing. Changes in emotional language, family-related language, and temporal orientation were explored as secondary outcomes.

In addition, we conducted an exploratory qualitative analysis of the narratives to examine recurrent experiential, relational, and identity-related features emerging during music-assisted recall, with particular attention to how participants communicated autobiographical scenes, meaningful roles, and emotionally salient life experiences.

We hypothesized that personalized music cues would (1) increase autobiographical narrative production, reflected in greater verbal output compared with baseline; and (2) be associated with increased use of cognitive-process and exclusion markers, consistent with greater reflective elaboration. Given the exploratory nature of the qualitative component, no a priori hypotheses were specified regarding thematic content. To test these aims, we compared baseline autobiographical narratives to music-assisted narratives analyzed via LIWC-22 within an exploratory repeated-measures pilot design, complemented by manual qualitative analysis of post-intervention narratives.

## 2. Materials and Methods

### 2.1. Sample and Description of Participants

Participants were recruited through convenience sampling from the Nursing Home Piccolo Cottolengo Don Orione (Seregno, Italy), a long-term residential care facility that provides medical, rehabilitative, and psychosocial support for older adults with varying levels of functional autonomy.

Inclusion criteria were the ability to provide written informed consent, adequate comprehension of Italian, and a Mini-Mental State Examination (MMSE) score > 19 (Folstein et al., 1975). This threshold was adopted to ensure sufficient cognitive functioning to understand study procedures and provide autonomous informed consent.

Exclusion criteria included a diagnosis of dementia, autism spectrum disorder, organic brain disease, and medical conditions likely to interfere with participation, such as severe mobility limitations preventing independent movement or significant language production difficulties that could compromise the ability to narrate autobiographical memories.

The study was conducted in accordance with the Declaration of Helsinki and was approved by the Ethics Committee of Università Cattolica del Sacro Cuore (protocol no. 87/25). All participants provided written informed consent prior to participation.

The final sample comprised 11 residents (9 men, 2 women), aged 65–86 years (M = 77.45, SD = 7.63). All participants had been residing in the nursing home for at least 10 years at the time of the study, indicating a shared long-term residential care context. As reported in Table 1, the mean adjusted MMSE score was 23.30 (SD = 4.06; range = 19.0–29.5). As a pilot study conducted within a residential care setting, sample size was determined by feasibility considerations rather than formal power analysis.

### 2.2. Intervention Structure and Autobiographical Recall Procedure

The study followed an exploratory within-subject repeated-measures pilot design over four weeks. During the baseline session, participants completed a single autobiographical interview without musical stimulation, during which they were invited to recall and narrate autobiographical memories from three distinct life periods: childhood, adolescence, and adulthood. Memory retrieval was elicited using standardized cue-word prompts derived from the Autobiographical Memory Test (AMT) procedure (Ros et al., 2018). Across both baseline and music-assisted sessions, participants were consistently asked: “What does the word *X* remind you of?” and were then invited to narrate a specific autobiographical memory associated with the cue word. For each life period, autobiographical recall was guided through three cue words selected from a predefined pool of age-related prompts. Cue words were presented in random order during the baseline session to minimize sequence effects. For childhood, the prompt pool included child, childhood, infancy, early age, carefree age, and early developmental years (Italian: bambino, fanciullezza, infanzia, tenera età, età della spensieratezza, età infantile). For adolescence, prompts included adolescence, youth, growing years, young adulthood, work, and formative years (Italian: adolescenza, gioventù, età della crescita, giovane adulto, lavoro, età della formazione). For adulthood, prompts included maturity, work, age of experience, age of awareness, achievement, and autonomy (Italian: età della maturità, lavoro, età dell’esperienza, età della consapevolezza, realizzazione, autonomia).

During the music-assisted sessions, different cue words from the same life-period pools were used to preserve comparability while reducing repetition effects. Interviewers minimized additional prompting and adopted a supportive, non-directive stance when participants experienced temporary difficulty recalling a memory. All autobiographical interviews across baseline and music-assisted conditions were conducted by the same interviewer for all participants in order to maximize procedural consistency and reduce interpersonal variability. The interviewer followed the same semi-structured procedure across all sessions, including standardized delivery of cue words and a consistent non-directive interviewing style. No procedural differences were introduced between baseline and music-assisted sessions in the manner or frequency of prompts. The baseline session provided a comprehensive pre-intervention linguistic profile.

No fixed time limit was imposed for responses to each cue word. Participants were encouraged to narrate freely at their own pace, in order to preserve the ecological and autobiographical nature of the task. The same standardized instruction was used across participants and sessions.

The intervention phase consisted of three weekly individual music-assisted sessions. Each session focused on one specific life period only (childhood, adolescence, adulthood), following the same developmental sequence explored at baseline (childhood first, then adolescence, then adulthood). During these sessions, participants first listened to their personalized playlist for approximately 10 min. Immediately afterward, they were presented with 3 cue words selected for evoking that life period, allowing for a comparable elicitation of autobiographical memories after musical stimulation.

The baseline session was conducted prior to the music sessions to obtain a pre-exposure autobiographical narrative sample uncontaminated by prior musical stimulation. The order of lifetime periods was kept constant across participants and was not counterbalanced. This choice was intended to preserve a coherent developmental structure and to reduce task demands in an older clinical population. However, the absence of counterbalancing and the fixed sequencing of sessions may have introduced order effects and should be considered when interpreting the findings.

Personalized playlists were developed through a semi-structured interview exploring preferred singers, musical genres, personally meaningful songs, and music associated with significant life periods or autobiographical memories. Based on these responses, a preliminary playlist was generated and subsequently refined in collaboration with the facility’s music therapist to ensure autobiographical relevance, familiarity, and clinical appropriateness. When discrepancies emerged, participant preferences were prioritized unless specific songs were considered potentially distressing or unsuitable within the care context. The final playlists are reported in the Supplementary Materials.

During the three music-assisted sessions, the same personalized playlist was used for each participant across sessions. However, the order of the tracks was varied between sessions to reduce repetition effects and maintain engagement. The full list of songs included in each participant’s playlist is reported in the Supplementary Materials.

All narratives were audio-recorded and transcribed verbatim in Italian. All transcripts were reviewed for accuracy prior to linguistic analysis. Formal inter-rater reliability coefficients were not computed.

### 2.3. Linguistic Analysis

To characterize the psychological and linguistic features of participants’ autobiographical narratives, transcripts were analyzed using Linguistic Inquiry and Word Count version 22 (LIWC-22) software (Boyd et al., 2022). LIWC is a validated computerized text analysis tool that quantifies the percentage of words in psychologically meaningful categories based on predefined dictionaries. The Italian built-in LIWC-22 dictionary was used for analysis to ensure linguistic and cultural appropriateness. LIWC computes the proportion of total words in each text that fall into specific linguistic and psychological categories, including affective processes (e.g., positive and negative emotion), social processes (e.g., family, communication), cognitive mechanisms (e.g., insight, causation), temporal orientation (past, present, future focus), and thematic domains (e.g., physical processes, mortality-related terms). LIWC employs a dictionary-based word-frequency approach and does not account for contextual or syntactic nuances. Findings therefore reflect aggregate linguistic patterns rather than semantic depth.

In this study, primary analyses focused on the following LIWC categories: total word count, cognitive processes, and exclusion words. Additional exploratory variables included positive emotion, negative emotion, family-related language, and past orientation.

In addition to the LIWC-based analysis, transcripts were reviewed qualitatively to identify recurrent phenomenological and relational features of music-assisted autobiographical recall. This component was intended as an illustrative, conceptually guided reading of the narratives rather than as a formal qualitative analysis.

### 2.4. Data Analysis

To explore potential changes in the linguistic characteristics of autobiographical narratives following the music intervention, Wilcoxon signed-rank tests were conducted for selected LIWC-22 categories and total word count. Primary analyses tested hypothesized changes in word count, cognitive-process language, and exclusion markers. Additional LIWC variables (positive emotion, negative emotion, family-related language, and past orientation) were examined exploratorily.

Given the small sample size and the percentage-based nature of LIWC variables, non-parametric tests were employed. Effect sizes (r) were calculated as r = Z/√N. To address the issue of multiple comparisons, the pattern of results was also examined using a false discovery rate (FDR) correction (Benjamini & Hochberg, 1995).

For both baseline and music-assisted conditions, narratives were elicited across three life periods (childhood, adolescence, adulthood). To ensure comparability across conditions, linguistic indices were computed by aggregating transcripts across the three sessions within each phase (pre-intervention and post-intervention). Aggregation was performed to reduce variability associated with specific life periods and to capture global changes in narrative structure and productivity. Analyses compared overall baseline autobiographical narratives with overall music-assisted autobiographical narratives at the participant level.

## 3. Results

Wilcoxon signed-rank tests were conducted to examine changes in linguistic characteristics of autobiographical narratives following the music intervention (N = 11). Results are summarized in Table 2. The increase in word count remained statistically significant after correction, while other comparisons did not reach significance.

### 3.1. Word Count

A Wilcoxon signed-rank test revealed a significant increase in total word count in the music-assisted condition relative to baseline, Z = −2.93, exact *p* < 0.001, r = 0.88.

**Figure 1:**
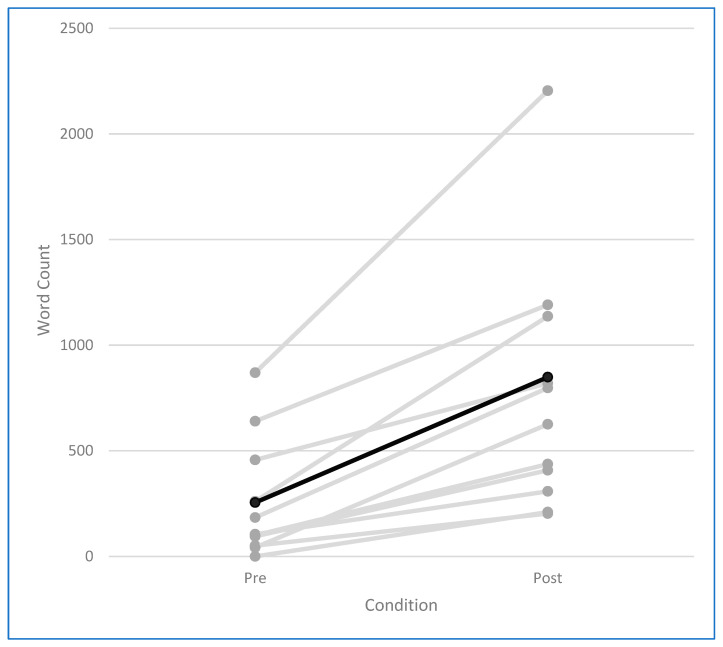
Individual trajectories of autobiographical narrative productivity (word count) from baseline to music-assisted condition. Each line represents one participant. The black line indicates the mean change across participants.

All participants showed an increase in word count from baseline to the music-assisted condition (Figure 1). This increase was observed across participants with varying levels of baseline cognitive functioning, including individuals with relatively lower MMSE scores.

#### Session-Level Descriptive Trends

**Figure 2:**
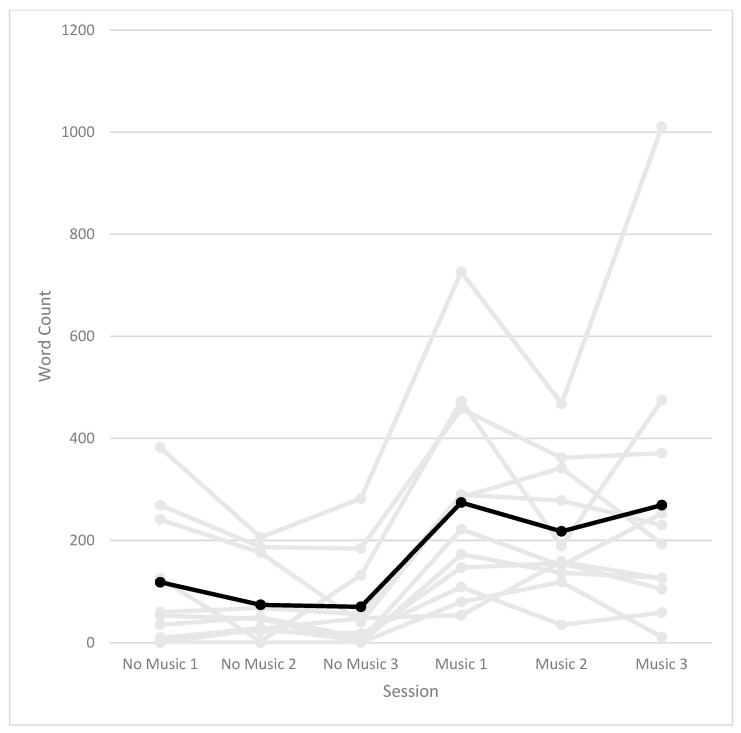
Individual trajectories of autobiographical narrative productivity (word count) across baseline (no music) and music-assisted sessions. Each line represents one participant. The black line indicates the mean trend across sessions.

To further explore potential order or practice effects, we examined descriptive changes in narrative productivity across individual sessions (Figure 2). As shown in the figure, word count did not exhibit a progressive increase across baseline (no music) sessions. Instead, narrative output showed a marked increase at the onset of the music-assisted condition and remained elevated across subsequent sessions. Although these observations are descriptive and should be interpreted cautiously, the session-level pattern alone cannot disentangle potential music-related effects from the structural differences between conditions. In particular, the transition from a single consolidated baseline session to dedicated post-intervention sessions for each life period may itself have contributed to the marked increase in narrative output.

### 3.2. Emotional Language

No statistically significant differences were observed in the proportional use of positive emotion words, Z = −0.66, exact *p* = 0.557, r = 0.21, or negative emotion words, Z = −0.89, exact *p* = 0.406, r = 0.28. However, descriptively, the median proportion of positive emotion words decreased from 2.96% at baseline to 0.54% in the music-assisted condition.

Importantly, LIWC variables are expressed as proportions of total word count. Given the substantial increase in total word count observed in the music-assisted condition, these proportional measures should be interpreted cautiously, as absolute frequencies of emotion-related words may have increased while remaining proportionally stable or even decreasing. Accordingly, the observed pattern may reflect dilution effects, inter-individual variability, or shifts in narrative focus, rather than a reliable reduction in positive emotional content.

### 3.3. Family-Related Language

No statistically significant differences were observed in the proportional use of family-related words, Z = −0.84, exact *p* = 0.461, r = 0.30. As LIWC variables are expressed as proportions of total word count, these findings should be interpreted cautiously in light of the substantial increase in overall narrative output.

### 3.4. Past Orientation

No statistically significant differences were observed in the proportional use of past-oriented words, Z = −1.48, exact *p* = 0.160, r = 0.47. As with other LIWC-derived measures, these results reflect proportional usage and should be interpreted cautiously given the increase in total word count.

### 3.5. Exclusion Language

The proportional use of exclusion-related words (contrastive markers) did not reach statistical significance, Z = −1.68, exact *p* = 0.105, r = 0.53. These findings should be interpreted cautiously, as proportional measures may not fully capture changes in absolute usage in the context of increased narrative length.

### 3.6. Cognitive Process Language

The proportional use of cognitive-process words did not reach statistical significance, Z = −1.78, exact *p* = 0.084, r = 0.56. As LIWC variables are based on relative frequencies, these results should be interpreted cautiously given the substantial increase in total word count across conditions.

### 3.7. Narrative Features of Music-Assisted Recall

In addition to the quantitative analyses, a qualitative paper-and-pencil analysis of the transcripts was conducted to identify recurrent experiential, narrative, and communicative patterns within the music-assisted autobiographical narratives. This qualitative component was exploratory and descriptive in nature and was intended to complement the linguistic findings by providing a clinically meaningful reading of how participants narrated their memories following personalized music listening. The manual review of the transcripts revealed four recurrent macro-areas that were consistently represented across participants:

(a) Re-emergence of concrete autobiographical scenes: many participants produced narratives characterized by vivid sensory or situational detail, suggesting that music may have facilitated access to autobiographical memories in scene-like form rather than as abstract summaries. For example, one participant recalled youthful experiences of singing outdoors with peers: “We used to go through the countryside… we sang along the road, among the fields… we were young girls… that was our happiness.” Another participant described childhood play with highly concrete bodily and environmental imagery: “We played hide-and-seek… we ran through the grass… we threw ourselves on the ground and rolled around.” Such excerpts suggest that music-assisted recall was often associated with the reconstruction of embodied and context-rich autobiographical moments rather than with generic life descriptions.
(b) Activation of identity and social belonging: several narratives indicated that songs were linked not only to private memories but also to shared identities, group membership, and collective emotional experiences. One participant described songs sung during football travel and tournament celebrations: “We sang Azzurro on the bus… then we sang We Are the Champions because that playoff day we won.” Another participant associated mountain songs with youth excursions, friendship, and vitality: “When we reached the refuge, we sang mountain songs… Sundays we all went to the mountains together.” These accounts suggest that personalized music may reactivate autobiographical memories embedded within broader social worlds, including friendship networks, leisure groups, sports communities, and generational culture.
(c) Recovery of meaningful former roles and self-images: in several cases, participants did not merely recall events but re-accessed meaningful former identities and life roles. One participant vividly described his youth as a gymnast, recalling competitions, specific apparatuses, teammates, and the pride of success. Other participants narrated themselves as workers, spouses, caregivers, or active community members. These narratives suggest that music-assisted recall may help reconnect individuals with temporally earlier versions of themselves that remain psychologically meaningful despite present institutional status.
(d) Emotional depth and increased readiness to communicate: the narratives frequently conveyed emotionally salient experiences, including love, grief, deprivation, family attachment, and loss. For example, one participant described the death of her husband in highly vivid terms and the continuing emotional significance of that event. Another spoke of the comfort derived from weekly visits by her son. Another explicitly stated that music had always offered “relief.”

## 4. Discussion

Narratives collected after personalized music exposure in this pilot sample of older adults living in nursing homes offer preliminary support for the idea that music may be associated with changes in how autobiographical memories (AMs) are narrated, particularly in relation to narrative productivity, communicative engagement, and access to personally meaningful material. Although the proportional distribution of lexical emotion terms remained largely unchanged, overall narrative output and several exploratory indices commonly interpreted as proxies for cognitive elaboration showed coherent post-exposure trends. Taken together, these findings suggest that the primary contribution of music may lie less in altering emotional valence at the word level and more in facilitating narrative activation and promoting a more productive autobiographical search. To our knowledge, this study is among the earliest to explore whether personalized music influences linguistic markers associated with autobiographical narration in older adults living in nursing homes, a group for whom autobiographical access and narrative self-continuity may be particularly vulnerable.

The most robust finding concerned narrative productivity, indexed by higher total word count in the music-assisted condition relative to baseline. This result should nevertheless be interpreted cautiously, as the post-intervention condition involved repeated sessions whereas baseline narratives were collected in a single session. Greater verbal output may therefore reflect not only music-related processes, but also increased familiarity with the task, repeated practice, or reduced cognitive load across sessions. Importantly, the baseline condition compressed autobiographical recall across all three life periods into a single session, whereas the post-intervention condition distributed these periods across separate dedicated sessions. This asymmetry in elicitation structure and available narrative space may itself have facilitated longer autobiographical accounts independently of any specific effect of music.

Even so, every participant produced a longer narrative after the intervention, yielding a very large effect. This pattern is consistent with enhanced engagement in autobiographical retrieval. Personalized music may function as a salient cue that facilitates access to associative networks linking songs with people, places, and life events (El Haj et al., 2013), likely because emotionally meaningful music recruits memory traces shaped by repeated pairings between songs and autobiographical contexts (Blood & Zatorre, 2001; Koelsch, 2014; El Haj et al., 2012; Janata et al., 2007).

Importantly, descriptive session-level patterns (see Figure 2) suggested that increases in narrative productivity were not gradual across baseline sessions but instead coincided with the onset of the music-assisted condition, although this observation should be interpreted cautiously.

The qualitative narratives suggest that this increase should not be reduced to mere verbosity. The music-assisted narratives frequently included autobiographical details such as names, places, sensory descriptions, temporal references, and emotionally meaningful episodes. Participants frequently reconstructed memories in scene-like form rather than as generic summaries, recalling for example childhood games, countryside singing with peers, journeys, competitions, or celebrations. This suggests that music may have enhanced not only the quantity of speech but also the availability of autobiographical material in a narratable form. In this sense, greater word count may reflect improved narrative accessibility and a greater willingness to remain engaged in remembering.

No statistically significant differences emerged in the proportional use of positive or negative emotion words. However, because LIWC indices are percentage-based measures, proportional stability does not necessarily imply that emotional language remained unchanged in absolute terms. Given the substantial increase in total word count, emotion-related words may also have increased in raw frequency while maintaining similar proportions. More broadly, the data suggest that music enhanced the accessibility and elaboration of autobiographical material while leaving its emotional composition relatively stable. Participants may therefore have narrated more, and perhaps more fully, without necessarily adopting a different emotional tone at the lexical level.

The qualitative material further reinforces this interpretation. Several narratives conveyed emotionally salient experiences involving love, grief, family attachment, deprivation, bereavement, and longing. One participant vividly described the death of her husband and the enduring emotional meaning of that loss; another emphasized the comfort derived from her son’s regular visits; another explicitly stated that music had always brought “relief.” These observations suggest that music-assisted recall facilitated access to meaningful experiences across the emotional spectrum. This interpretation is consistent with evidence that music-evoked AMs are often emotionally intense yet not systematically biased toward positive content (Jakubowski & Eerola, 2022). Personalized music may therefore enrich recall through mnemonic activation and experiential vividness rather than by inducing a generalized positivity shift.

More nuanced effects emerged in exploratory linguistic indices potentially related to reflective or contrastive processing. Although these did not reach conventional statistical significance, both exclusion markers and cognitive-process words showed moderate-to-large effect sizes, with directional increases for most participants. These categories have been associated with narrative differentiation, evaluative processing, reappraisal, and meaning-making processes that go beyond simple episodic listing (Pennebaker et al., 2015). While caution is warranted, the overall pattern may tentatively indicate a shift from predominantly descriptive recall toward a more reflective and integrative narrative mode, an aspect central to narrative identity formation and maintenance (Frisone et al., 2025; Stramba-Badiale et al., 2025).

The qualitative findings again help contextualize this pattern. Several participants moved beyond recounting events and introduced broader reflections, values, and interpretative statements about life, aging, loss, or resilience. Others contrasted past and present circumstances or re-evaluated earlier experiences from a current perspective. Such discursive movements are consistent with the idea that music may support not only retrieval of memories but also a more elaborative stance toward autobiographical material.

From a theoretical standpoint, these trends can be situated within models of AM such as the Self-Memory System, which conceptualizes autobiographical recall as a hierarchical structure integrating episodic details with broader life-story knowledge and self-schemas (Conway & Pleydell-Pearce, 2000; Conway, 2005). Personalized music may facilitate access to this system by activating emotionally salient associative networks that serve as efficient entry points into autobiographical knowledge, thereby supporting more elaborated retrieval and stronger cognitive scaffolding during narration. In this framework, the observed directional increase in cognitive-process language may reflect processes akin to life review, which emphasizes reflective integration of experiences and the construction of coherence rather than mere chronological enumeration (Frisone et al., 2024a, 2024b; Westerhof et al., 2010).

The qualitative narratives also suggest that music may reactivate identity positions that are often obscured in residential care settings. Participants did not appear only as current residents of a nursing home, but re-emerged as gymnasts, workers, spouses, daughters, caregivers, football supporters, travelers, or young friends. This suggests that music may reactivate not only personal memory, but also a sense of belonging to meaningful social worlds. In long-term care contexts, where interactions may become centered on routines and care needs, such processes may help preserve narrative continuity and reinforce personhood beyond present dependency status (McLean et al., 2007).

Temporal orientation also showed a moderate tendency toward reduced past-tense markers. Although speculative, this finding may be compatible with increased experiential immediacy during narration, consistent with ‘mental time travel’ accounts of episodic remembering (Tulving, 2002). Music-evoked AMs have been associated with heightened vividness and immersion (Janata et al., 2007), which could influence linguistic framing by encouraging a more phenomenological mode of recounting, even when events remain temporally anchored in the past.

Overall, the present findings suggest that personalized music listening may influence autobiographical narration primarily by enhancing linguistic, relational and productivity-related features, rather than by directly modifying emotional content at the lexical level. This distinction matters theoretically and clinically: in older adults living in nursing homes, maintaining narrative coherence and a sense of self-continuity can be closely tied to psychological well-being, identity stability, and adaptive coping (Addis & Tippett, 2004; de Medeiros et al., 2020). If music supports narrative activation and elaboration, it may offer a mechanism for strengthening self-relevant remembering even when the emotional tone of recalled material remains mixed or unchanged. Such an account also aligns with the idea that music may preferentially facilitate experiential immersion and integrative processing, rather than promoting a simple positivity effect (Rossi et al., 2024b). More broadly, the value of personalized music in residential care may lie not only in memory stimulation, but also in reopening emotionally meaningful communicative space through which identity can still be expressed, recognized, and shared.

### Limitations and Future Direction

The findings of this exploratory pilot study must be interpreted within several methodological constraints. First, the absence of a control condition prevents firm causal conclusions. Without a comparison group (e.g., non-musical reminiscence or passive listening), it is not possible to disentangle the specific effects of personalized music from potential practice effects, repeated narrative elicitation, or interviewer-related influences.

Second, linguistic analysis relied on dictionary-based word-frequency measures. Although LIWC provides a validated and objective approach to quantifying psychologically relevant language patterns, it does not capture contextual meaning, narrative coherence, syntactic complexity, or higher-order discourse organization. Accordingly, the present findings should be interpreted as lexical indicators associated with psychological and communicative processes rather than direct measures of narrative integration.

Third, post-intervention narratives were elicited across different life phases. Although this developmentally structured approach was theoretically grounded, it may have introduced variability related to the emotional salience or accessibility of specific autobiographical periods.

Finally, the sample was small, predominantly male, and recruited from a single nursing home facility, limiting external validity. Although all participants shared a long-term residential context (at least 10 years of residence), demographic, cultural, and institutional factors may influence music preferences, emotional expressivity, autobiographical style, and responsiveness to music-based interventions. In particular, the predominance of male participants may have influenced verbal productivity, emotional language use, and autobiographical narrative style, as these domains may vary across gender and cohort experiences. Baseline cognitive functioning may also moderate responsiveness to music cues and narrative output. In the present sample, participants across the observed MMSE range (19.0–29.5) generally showed increases in narrative output following the music-assisted condition. However, given the small sample size, these observations are descriptive only and no reliable conclusions can be drawn regarding potential moderation by cognitive functioning. Larger studies should examine whether MMSE or other cognitive measures predict intervention-related change.

Future research should therefore employ larger and more diverse samples, multi-site recruitment, and randomized controlled designs comparing personalized music with appropriate control conditions. Integrating LIWC with more advanced natural language processing approaches capable of capturing contextual and discourse-level features may further clarify how music influences autobiographical remembering and narrative expression in later life.

## 5. Conclusions

Despite the limitations of the present study, the convergence between higher narrative output in the post-intervention condition and the consistent directional patterns observed in exploratory linguistic markers suggests that personalized music may represent a promising avenue for supporting autobiographical expression in nursing home residents. Although the current design does not allow firm causal conclusions, the findings are consistent with the possibility that personalized music may help facilitate engagement with autobiographical remembering and narrative expression in later life.

Taken together, these preliminary findings provide a rationale for further investigation in larger and methodologically rigorous studies. Future research should prioritize adequately powered and controlled designs to determine whether personalized music can reliably enhance autobiographical narration beyond the effects of repeated recall, session structure, or interpersonal familiarity. Clarifying these mechanisms may help define the role of personalized music as a low-cost, human-centered resource for sustaining identity and communication in residential care.

## Figures and Tables

**Table 1:** Demographic and Clinical Characteristics of Participants.

Variable	n (%)	M (SD)	Range
Age (years)	—	77.45 (7.63)	65–86
Sex			
Male	9 (81.8%)	—	—
Female	2 (18.2%)	—	—
Adjusted MMSE	—	23.30 (4.06)	19.0–29.5

Note. MMSE = Mini-Mental State Examination (adjusted score).

**Table 2:** Pre–Post Comparisons of Linguistic Variables (Wilcoxon Signed-Rank Tests).

Variable	Median Pre	Median Post	Z	Exact *p*	r
Word Count	105.00	626.00	−2.93	<0.001	0.88
Positive Emotion	2.96	0.54	−0.66	0.557	0.21
Negative Emotion	1.81	1.43	−0.89	0.406	0.28
Family	1.04	0.80	−0.84	0.461	0.30
Past Orientation	9.58	8.30	−1.48	0.160	0.47
Exclusion	2.58	4.35	−1.68	0.105	0.53
Cognitive Processes	2.40	2.96	−1.78	0.084	0.56

Note. *p*-values are two-tailed exact probabilities from Wilcoxon signed-rank tests. Effect sizes were calculated as r = Z/N. To control for multiple comparisons across the seven linguistic variables, a false discovery rate (FDR) correction was applied using the Benjamini–Hochberg procedure (Benjamini & Hochberg, 1995). Only the word count comparison remained statistically significant after correction.

## Data Availability

The raw data supporting the conclusions of this article will be made available by the authors on request.

## Supplementary Materials

The following supporting information can be downloaded at: https://www.mdpi.com/article/10.3390/bs16050810/s1, Table S1: Playlists used in the study.
