# Supplementary material for: Personalized Music Listening and Autobiographical Narration in Nursing Home Residents: Linguistic and Qualitative Findings from a Pilot Study
Source: Behav Sci (Basel). 2026 May 19;16(5):810. doi: 10.3390/bs16050810 (PMC13203300; doi:10.3390/bs16050810)
Supplement: Supplementary file 1 [file behavsci-16-00810-s001.zip › behavsci-4215139-supplementary.pdf]

Supplementary Material

Table S1. Playlists used in the study

ID	Preferences	Playlist
**1**	Mountain folk music, Gianni Morandi, Orietta Berti	1. *Vecchio scarpone* – Traditional song<br>2. *Sul cappello* – Traditional Alpine song<br>3. *Fatti mandare dalla mamma* – Gianni Morandi
**2**	Adriano Celentano, Jean Sibelius, Giacomo Puccini, Giuseppe Verdi, Frederick Chopin	1. *Azzurro* – Adriano Celentano<br>2. *Valse triste op.44* – Jean Sibelius<br>3. *Coro a bocca chiusa* (da *Madama Butterfly*) – Giacomo Puccini
**3**	Folk Music, Gianni Morandi, Adriano Celentano, Orietta Berti	1. *Quel mazzolin di fiori* – Traditional song<br>2. *Fin che la barca va* – Orietta Berti<br>3. *Romagna mia* – Secondo Casadei
**4**	Fred Bongusto, Bobby Solo, Gianni Morandi, Little Tony, Nicola Di Bari	1. *Una lacrima sul viso* – Bobby Solo<br>2. *Una rotonda sul mare* – Fred Bongusto<br>3. *Riderà* – Little Tony
**5**	Collage, Lucio Battisti, Pooh, Cugini di campagna	1. *Due ragazzi nel sole* – Collage<br>2. *Piccola Katy* – Pooh<br>3. *Anima mia* – Cugini di campagna
**6**	Laura Pausini, Gianni Morandi, Adriano Celentano	1. *Strani amori* – Laura Pausini<br>2. *Fatti mandare dalla mamma* – Gianni Morandi<br>3. *Azzurro* – Adriano Celentano
**7**	Vasco Rossi, Gianni Morandi	1. *Fatti mandare dalla mamma* – Gianni Morandi<br>2. *Ogni volta* – Vasco Rossi<br>3. *Andavo a cento all'ora* – Gianni Morandi
**8**	Pooh, Folk Music, La banda d'Affori, Biagio Antonacci	1. *Quel mazzolin di fiori* – Traditional song<br>2. *I pompieri di Viggiù* – Traditional song<br>3. *Il tamburo della banda d'Affori* – Traditional song *Tanta voglia di lei* – Pooh

ID	Preferenze	Canzoni ascoltate
**9**	Umberto Tozzi, Gianni Morandi, Enrico Ruggeri, Max Gazzé, Adriano Celentano	1. *Azzurro* – Adriano Celentano<br>2. *Si può dare di più* – Gianni Morandi, Enrico Ruggeri, Umberto Tozzi<br>3. *Gloria* – Umberto Tozzi
**10**	Folk Music, Orietta Berti, Albano, Romina, Mino Reitano	1. *Quel mazzolin di fiori* – Traditional song<br>2. *Mamma dammi cento lire* – Le mondine<br>3. *Vecchio scarpone* – Traditional song
**11**	Gianni Morandi, Adriano Celentano, Albano e Romina, Folk Music	1. *Romagna mia* – Secondo Casadei<br>2. *Quel mazzolin di fiori* – Traditional song<br>3. *Azzurro* – Adriano Celentano
